# Crystal structure, Hirshfeld surface analysis, crystal voids, inter­action energy calculations and energy frameworks and DFT calculations of ethyl 2-cyano-3-(3-hy­droxy-5-methyl-1*H*-pyrazol-4-yl)-3-phen­yl­propano­ate

**DOI:** 10.1107/S2056989024000744

**Published:** 2024-01-31

**Authors:** Younesse Ait Elmachkouri, Ezaddine Irrou, Hanae El Monfalouti, Ahmed Mazzah, Tuncer Hökelek, Joel T. Mague, Mohamed Labd Taha, Nada Kheira Sebbar

**Affiliations:** a Laboratory of Organic and Physical Chemistry, Applied Bioorganic Chemistry Team, Faculty of Sciences, Ibn Zohr University, Agadir, Morocco; b Laboratory of Plant Chemistry, Organic and Bioorganic Synthesis, Faculty of Sciences, Mohammed V University in Rabat, 4 Avenue Ibn Battouta BP 1014 RP, Morocco; c University of Lille, CNRS, UAR 3290, MSAP, Miniaturization for Synthesis, Analysis and Proteomics, F-59000 Lille, France; d Department of Physics, Hacettepe University, 06800 Beytepe, Ankara, Türkiye; e Department of Chemistry, Tulane University, New Orleans, LA 70118, USA; Universidad de la Repüblica, Uruguay

**Keywords:** crystal structure, pyrazole, C—H⋯π(ring) inter­action, hydrogen bond

## Abstract

The title compound, C_16_H_17_N_3_O_3_, is racemic as it crystallizes in a centrosymmetric space group (*P*  ), although the *trans* disposition of substituents about the central C—C bond is established. The five- and six-membered rings are oriented at a dihedral angle of 75.88 (8)°. In the crystal, N—H⋯N hydrogen bonds form chains of mol­ecules extending along the *c*-axis direction that are connected by inversion-related pairs of O—H⋯N into ribbons. The ribbons are linked by C—H⋯π(ring) inter­actions, forming layers parallel to the *ab* plane. A Hirshfeld surface analysis indicates that the most important contributions for the crystal packing are from H⋯H (45.9%), H⋯N/N⋯H (23.3%), H⋯C/C⋯H (16.2%) and H⋯O/O⋯H (12.3%) inter­actions. Hydrogen bonding and van der Waals inter­actions are the dominant inter­actions in the crystal packing. The volume of the crystal voids and the percentage of free space were calculated to be 100.94 Å^3^ and 13.20%, showing that there is no large cavity in the crystal packing. Evaluation of the electrostatic, dispersion and total energy frameworks indicates that the stabilization is dominated by the electrostatic energy contributions in the title compound. Moreover, the DFT-optimized structure at the B3LYP/6–311 G(d,p) level is compared with the experimentally determined mol­ecular structure in the solid state. The HOMO–LUMO behaviour was elucidated to determine the energy gap.

## Abstract 2

In the title mol­ecule, the five- and six-membered rings are oriented at a dihedral angle of 75.88 (8)°. In the crystal, N—H⋯N hydrogen bonds form chains of mol­ecules extending along the *c*-axis direction that are connected by inversion-related pairs of O—H⋯N into ribbons. The ribbons are linked by C—H⋯π(ring) inter­actions, forming layers parallel to the *ab* plane.

## 1. Chemical context

As part of our ongoing investigation into the use of pyrazoles to develop new heterocyclic systems (Moukha-Chafiq *et al.*, 2006 ▸; Elmachkouri *et al.*, 2022 ▸; Moukha-Chafiq *et al.*, 2007*a* ▸; Irrou *et al.*, 2022 ▸), particularly those likely to exhibit intriguing biological activities, we note that compounds sharing structural similarities with pyrazole have demonstrated potential in various biological domains, exhibiting analgesic (Gursoy *et al.*, 2000 ▸), anti­fungal and anti­bacterial (Prasath *et al.*, 2015 ▸; Akbas *et al.*, 2005 ▸), anti­viral (Moukha-Chafiq *et al.*, 2007*b* ▸) and anti­cancer (Bensaber *et al.*, 2014 ▸) activities. Consequently, the development of innovative synthetic pathways aims to obtain new mol­ecules with structures that are better adapted to cellular receptors. In this respect, we recently reported the synthesis of some pyran­opyrazoles (Ait Elmachkouri *et al.*, 2023*a* ▸) and pyrazolo­pyran­opyrimidines (Ait Elmachkouri *et al.*, 2023*b* ▸). In our ongoing research, we focus our inter­est on pyrazole derivatives and present there the synthesis of ethyl 2-cyano-3-(3-hy­droxy-5-methyl-1*H*-pyrazol-4-yl)-3-phenyl­propano­ate, (I). For this synthesis, we adopted a three-component approach, using 3-methyl-1*H*-pyrazol-5-ol, ethyl 2-cyano­acetate and benzaldehyde in ethanol in the presence of piperidine as base. Additionally, we conducted a Hirshfeld surface analysis and performed calculations on inter­molecular inter­action energies and energy frameworks. We compared the mol­ecular structure optimized using density functional theory (DFT) at the B3LYP/6-311G(d,p) level, with the experimentally determined mol­ecular structure in its solid state. 
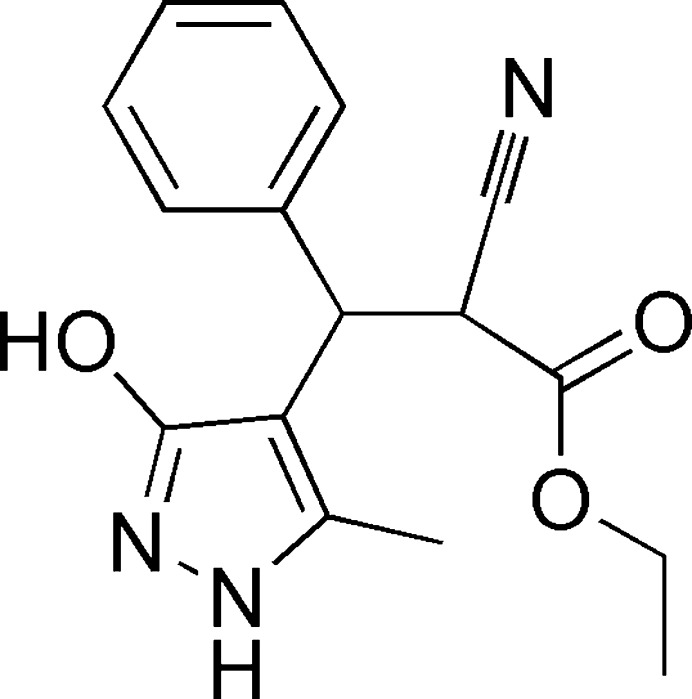


## 2. Structural commentary

**Figure 1:**
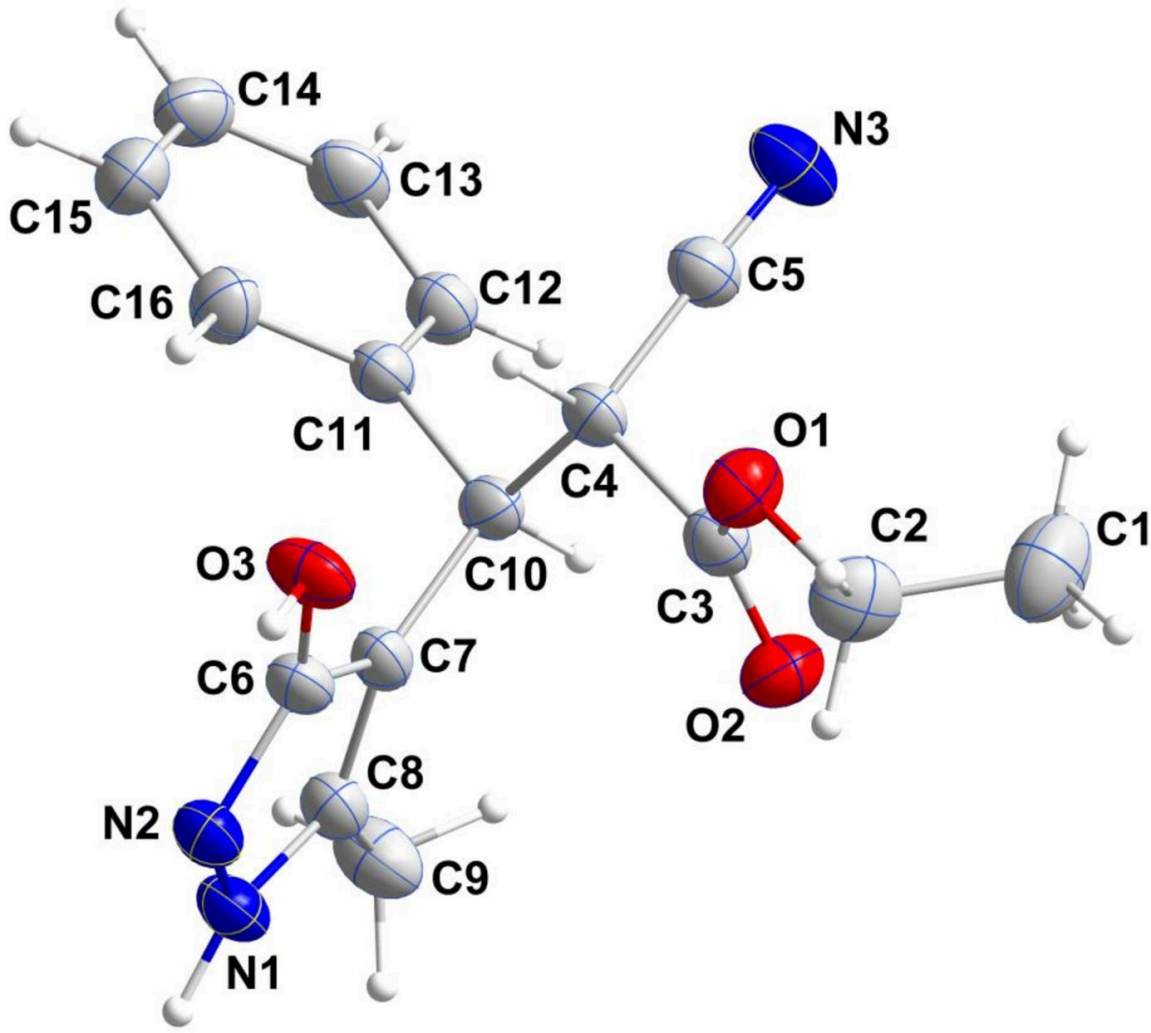
Perspective view of the title mol­ecule with labelling scheme and 50% probability ellipsoids.

As the title compound (I), (Fig. 1 ▸) crystallizes in a centrosymmetric space group (*P* 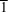 ), the sample is racemic although the *trans* disposition of substituents about the C4—C10 bond is established. The dihedral angle between the mean planes of the five- and six-membered rings is 75.88 (8)°, while the sum of the angles about N1 is 360° within experimental error, implicating involvement of its lone pair in intra-ring π bonding. The rotational orientation of the five-membered ring may be partially determined by a C4—H4⋯O3 hydrogen bond (H4⋯O3 = 2.41 Å) although the C4—H4⋯O3 angle of 115° is quite small for such an inter­action.

## 3. Supra­molecular features

**Figure 2:**
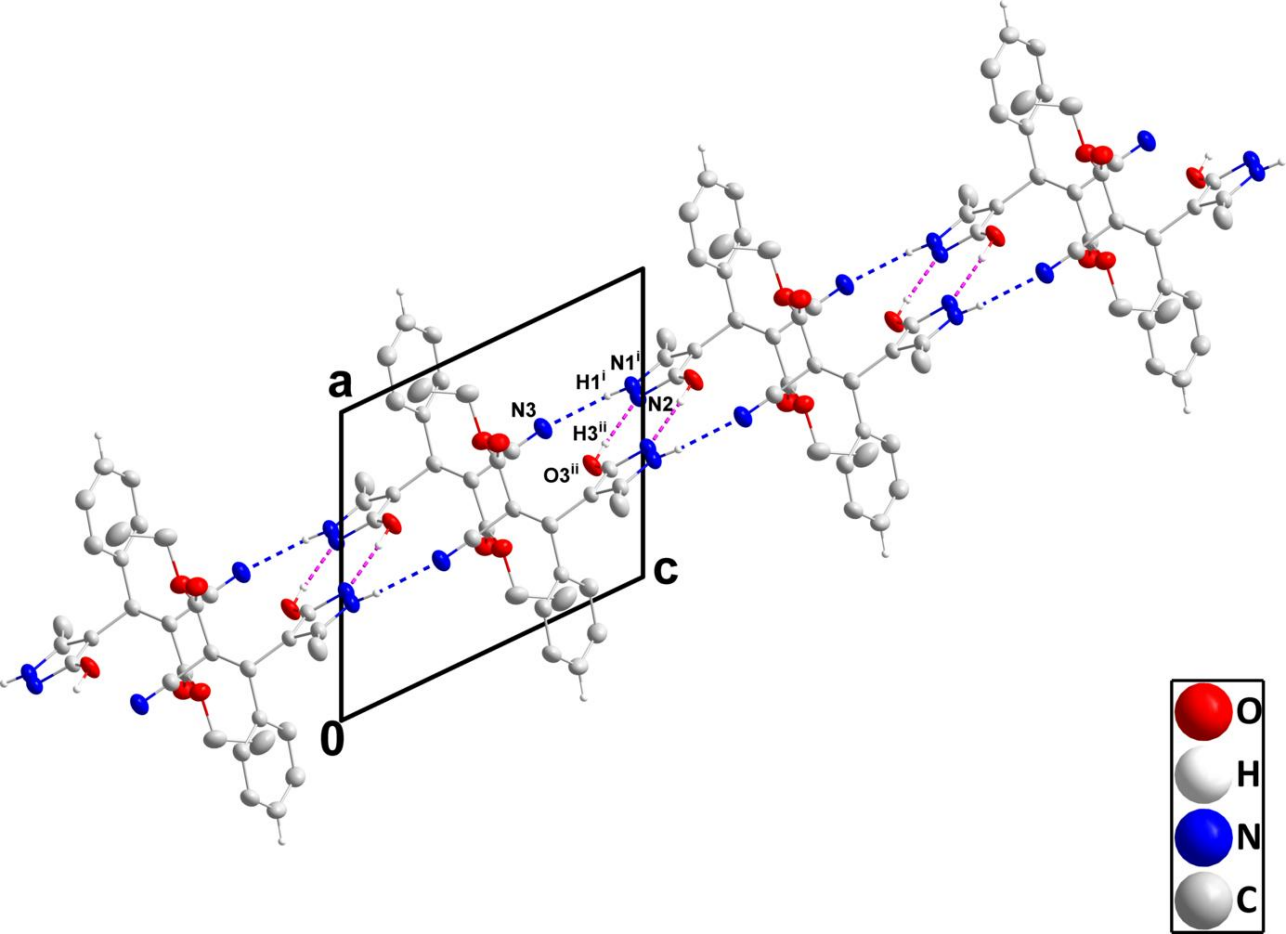
A portion of one ribbon viewed along the *b*-axis direction with N—H⋯N and O—H⋯N hydrogen bonds depicted, respectively, by blue and dark-pink dashed lines. Hydrogen atoms not involved in these inter­actions are omitted for clarity. [Symmetry codes: (i) *x*, *y*, *z* + 1; (ii) −*x* + 1, −*y* + 2, −*z* + 1.]

**Figure 3:**
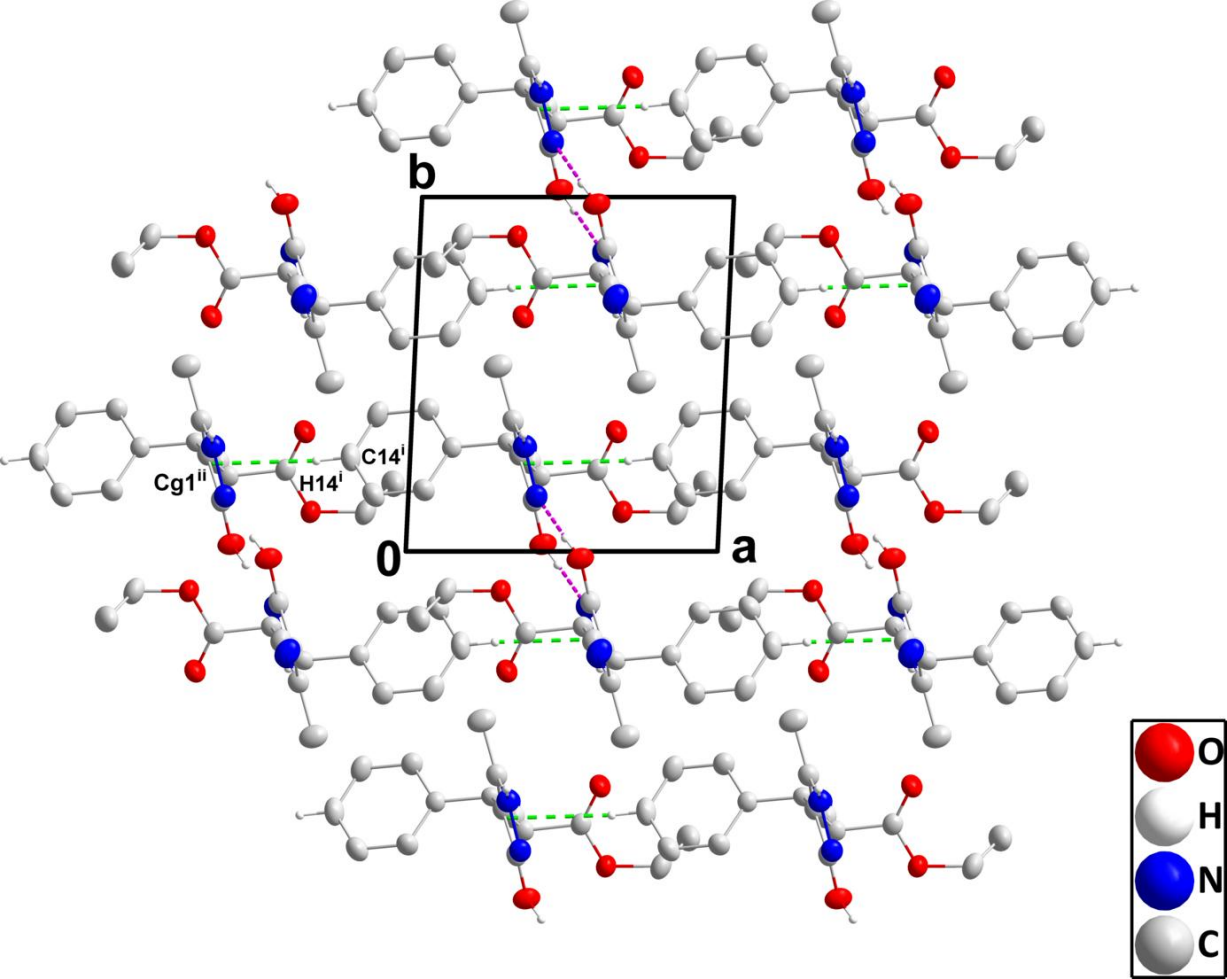
Packing viewed along the *b*-axis direction with N—H⋯N and O—H⋯N hydrogen bonds depicted, respectively, by blue and dark-pink dashed lines while the C—H⋯π(ring) inter­actions are depicted by green dashed lines. Hydrogen atoms not involved in these inter­actions are omitted for clarity. [Symmetry codes: (i) −*x* + 1, −*y* + 1, −*z* + 1; (ii) *x* − 1, *y* − 1, *z*).

In the crystal, N1—H1⋯N3 hydrogen bonds (Table 1 ▸) form chains of mol­ecules extending along the *c*-axis direction that are connected by inversion-related pairs of O3—H3⋯N2 hydrogen bonds into ribbons (Fig. 2 ▸). The ribbons are linked by C14—H14⋯*Cg*1 inter­actions (Table 1 ▸), forming layers parallel to the *ab* plane (Fig. 3 ▸).

## 4. Hirshfeld surface analysis

**Figure 4:**
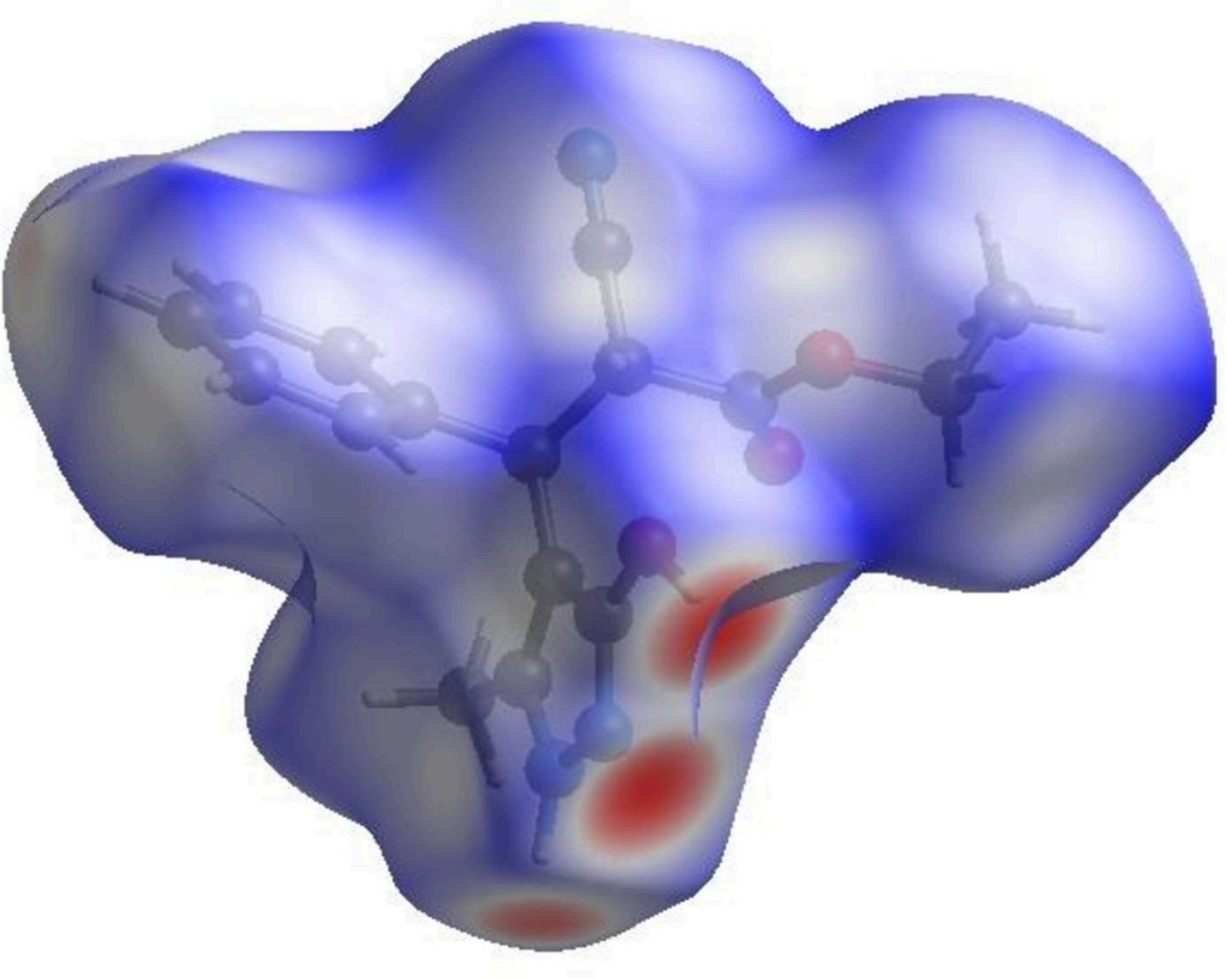
View of the three-dimensional Hirshfeld surface of the title compound plotted over *d*_norm_.

**Figure 5:**
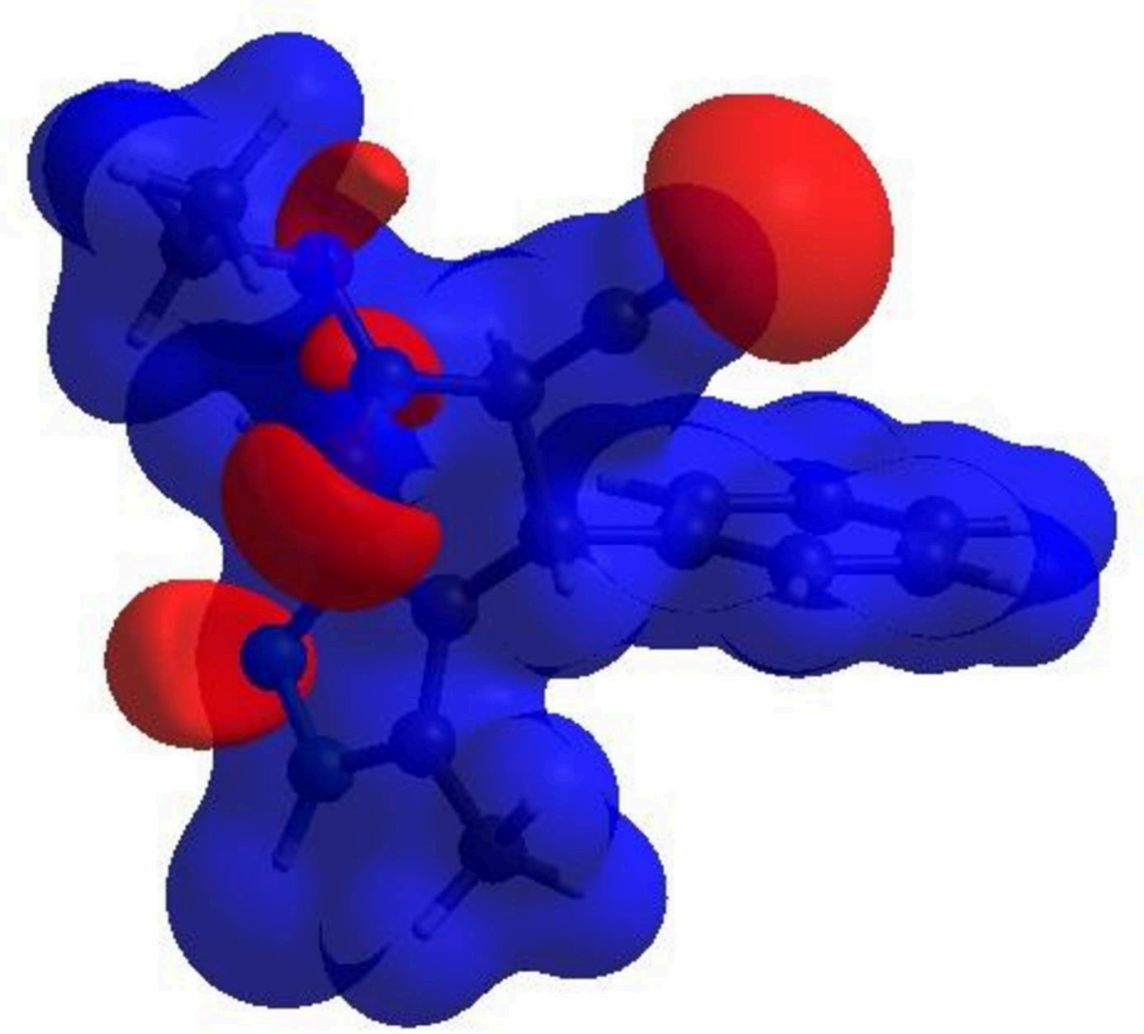
View of the three-dimensional Hirshfeld surface of the title compound plotted over electrostatic potential energy using the STO-3 G basis set at the Hartree–Fock level of theory. Hydrogen-bond donors and acceptors are shown as blue and red regions, respectively, around the atoms corresponding to positive and negative potentials.

**Figure 6:**
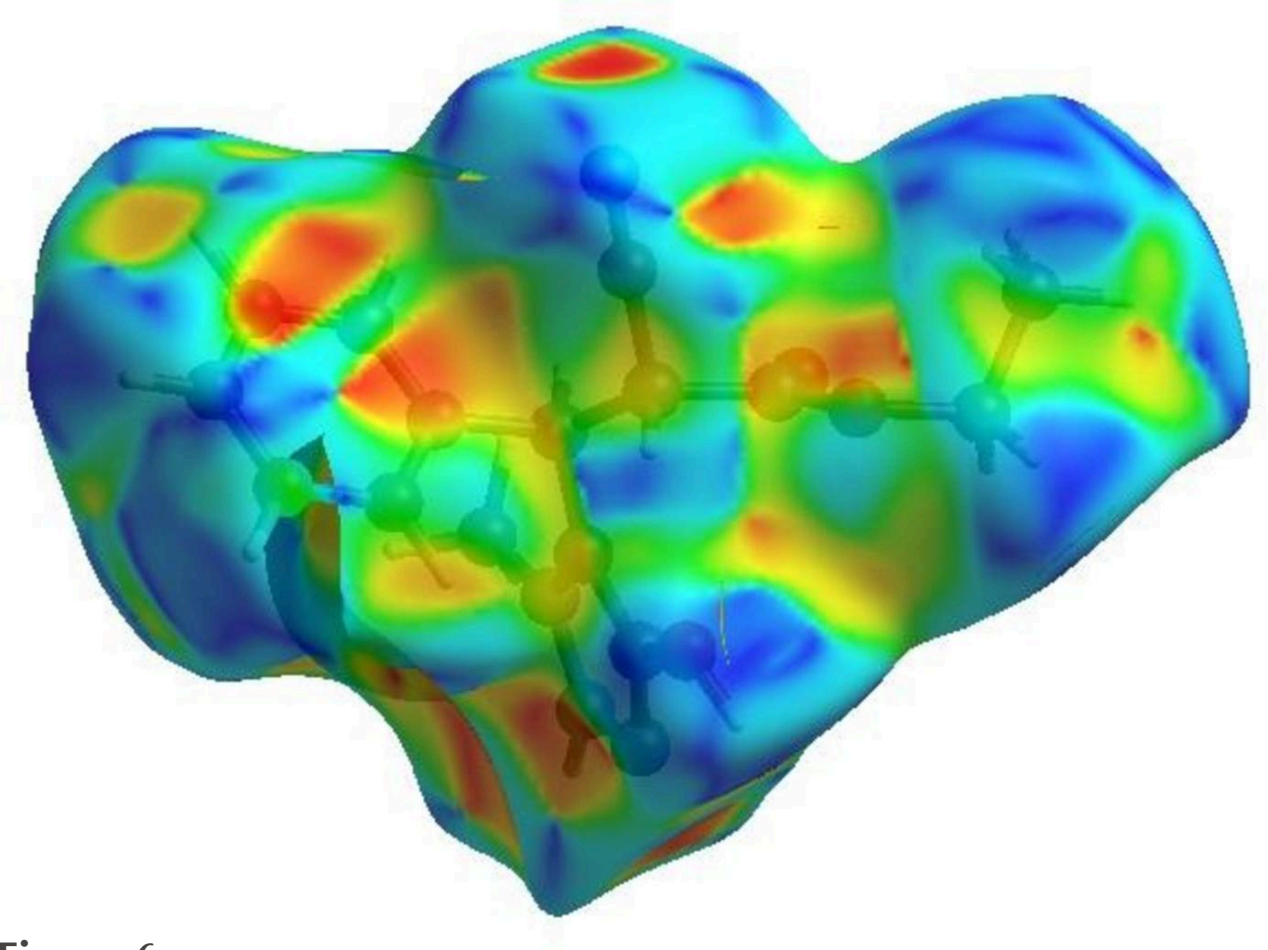
Hirshfeld surface of the title compound plotted over shape-index.

In order to visualize the inter­molecular inter­actions in the crystal of the title compound (I), a Hirshfeld surface (HS) analysis (Hirshfeld, 1977 ▸; Spackman & Jayatilaka, 2009 ▸) was carried out using *Crystal Explorer 17.5* (Turner *et al.*, 2017 ▸). In the HS plotted over *d*_norm_ (Fig. 4 ▸), the white surface indicates contacts with distances equal to the sum of van der Waals radii, and the red and blue colours indicate distances shorter (in close contact) or longer (distant contact) than the van der Waals radii, respectively (Venkatesan *et al.*, 2016 ▸). The bright-red spots indicate their roles as the respective donors and/or acceptors; they also appear as blue and red regions corres­ponding to positive and negative potentials on the HS mapped over electrostatic potential (Spackman *et al.*, 2008 ▸; Jayatilaka *et al.*, 2005 ▸) shown in Fig. 5 ▸. The blue regions indicate positive electrostatic potential (hydrogen-bond donors), while the red regions indicate negative electrostatic potential (hydrogen-bond acceptors). The shape-index of the HS is a tool to visualize π–π stacking by the presence of adjacent red and blue triangles; if there are no adjacent red and/or blue triangles, then there are no π–π inter­actions. Fig. 6 ▸ clearly suggests that there are no π–π inter­actions in (I).

**Figure 7:**
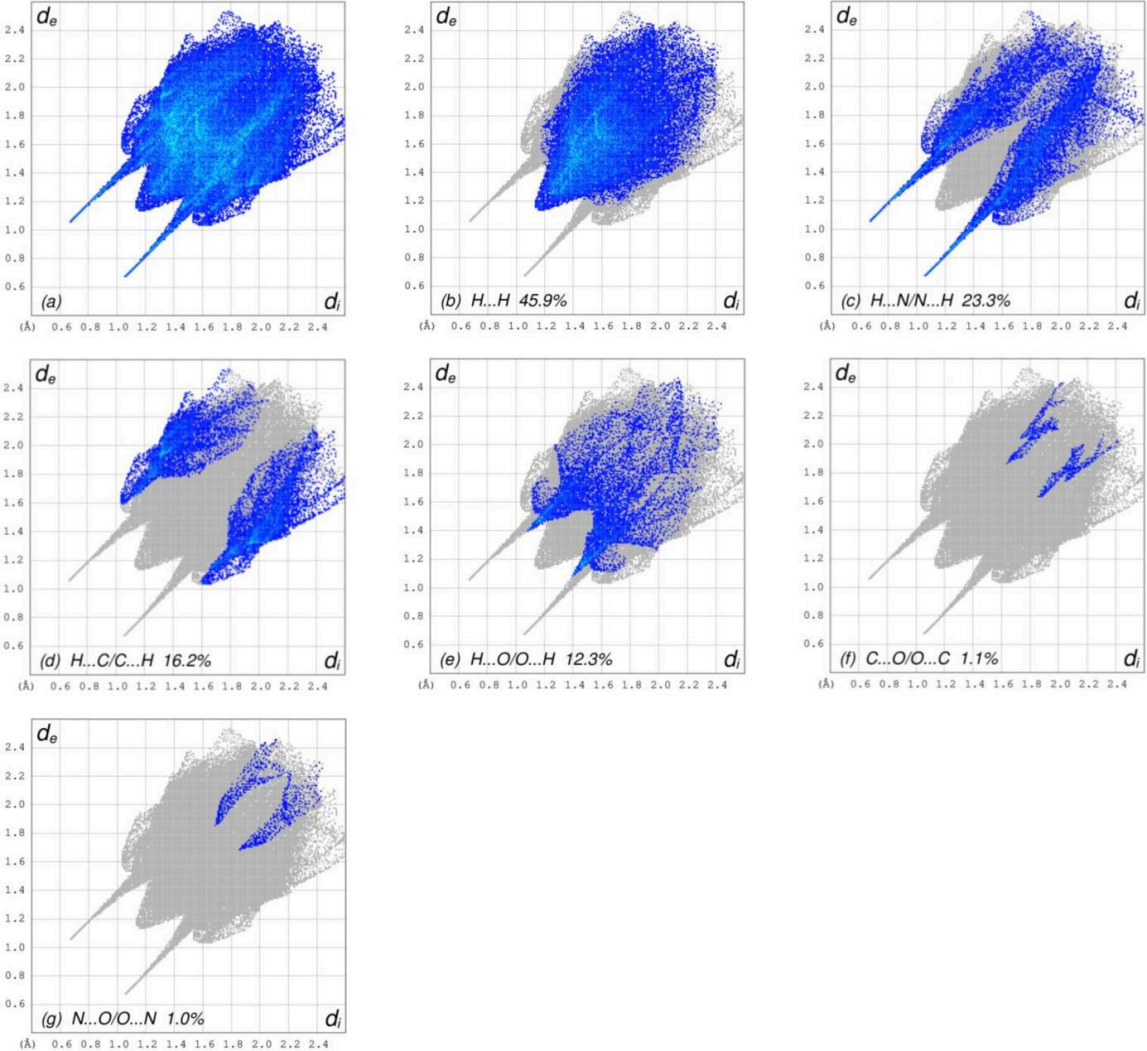
The full two-dimensional fingerprint plots for the title compound, showing (*a*) all inter­actions, and delineated into (*b*) H⋯H, (*c*) H⋯N/N⋯H, (*d*) H⋯C/C⋯H, (*e*) H⋯O/O ⋯ H, (*f*) C⋯O/O⋯C and (*g*) N⋯O/O⋯N inter­actions. The *d*_i_ and *d*_e_ values are the closest inter­nal and external distances (in Å) from given points on the Hirshfeld surface.

The overall two-dimensional fingerprint plot, Fig. 7 ▸ *a*, and those delineated into H⋯H, H⋯N/N⋯H, H⋯C/C⋯H, H⋯O/O⋯H, C⋯O/O⋯C and N⋯O/O⋯N inter­actions (McKinnon *et al.*, 2007 ▸) are illustrated in Fig. 7 ▸ *b*–*g* respectively, together with their relative contributions to the Hirshfeld surface. The most abundant inter­action is H⋯H, contributing 45.9% to the overall crystal packing, which is reflected in Fig. 7 ▸ *b* as widely scattered points of high density due to the large hydrogen content of the mol­ecule with the tip at *d*_e_ = *d*_i_ = 1.15 Å. The symmetrical pair of spikes in the fingerprint plot delineated into H⋯N/N⋯H contacts (Fig. 7 ▸ *c*), with a 23.3% contribution to the HS, has the tips at *d*_e_ + *d*_i_ = 1.72 Å. In the presence of C—H⋯π inter­actions, the H⋯C/C⋯H contacts, contributing 16.2% to the overall crystal packing, Fig. 7 ▸ *d*, have the tips at *d*_e_ + *d*_i_ = 2.64 Å. The symmetrical pair of spikes in the fingerprint plot delineated into H⋯O/O⋯H contacts (Fig. 7 ▸ *e*, 12.3% contribution to the HS) has the tips at *d*_e_ + *d*_i_ = 2.48 Å. Finally, the C⋯O/O⋯C (Fig. 7 ▸ *f*) and N⋯O/O⋯N (Fig. 7 ▸ *g*) contacts with 1.1% and 1.0% contributions, respectively, to the HS have a very low distribution of points.

**Figure 8:**
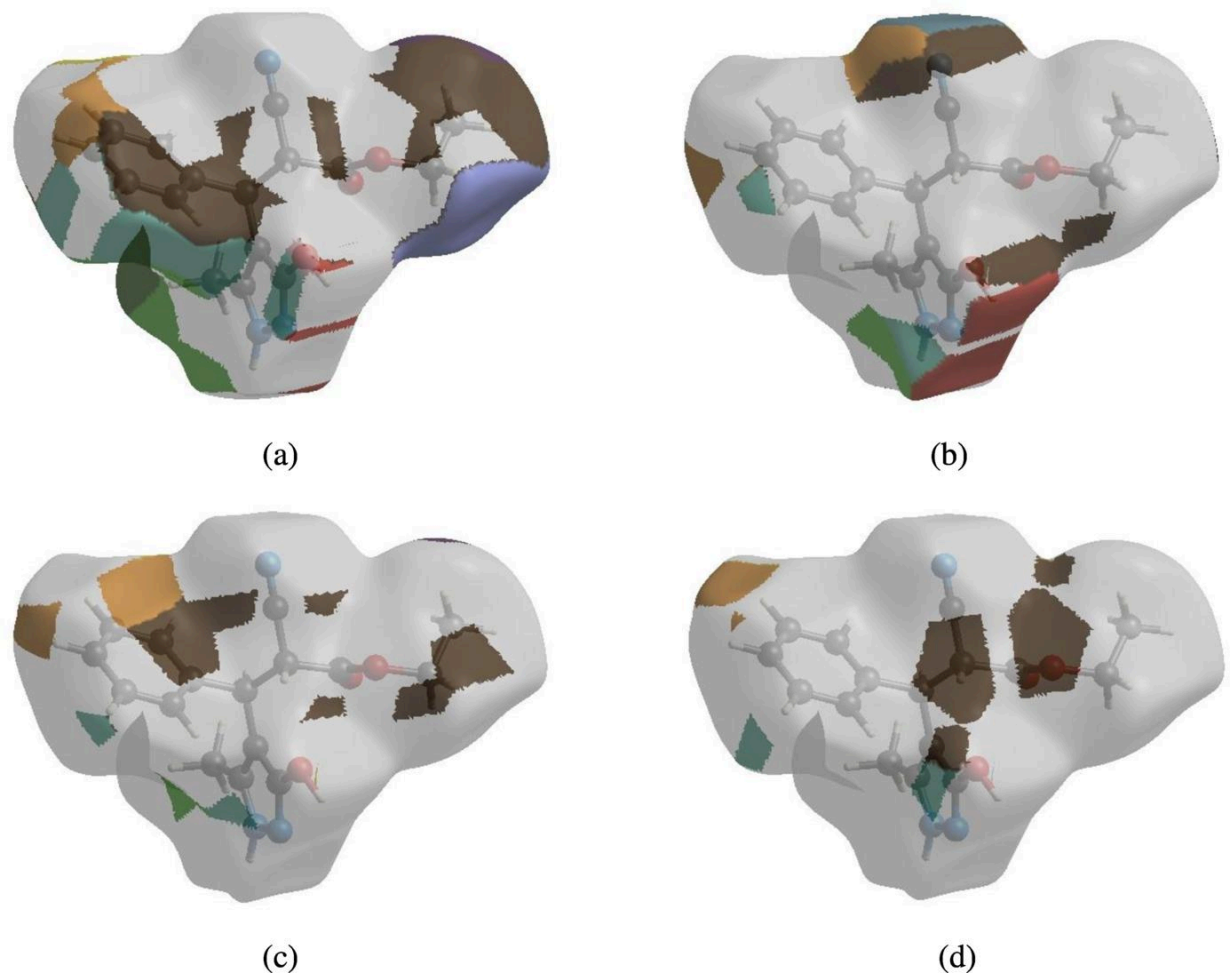
The Hirshfeld surface representations with the function fragment patch plotted onto the surface for (*a*) H⋯H, (*b*) H⋯N/N⋯H, (*c*) H ⋯ C/C⋯H and (*d*) H⋯O/O⋯H inter­actions.

The nearest neighbour coordination environment of a mol­ecule can be determined from the colour patches on the HS based on how close to other mol­ecules they are. The Hirshfeld surface representations with the function *d*_norm_ plotted onto the surface are shown for the H⋯H, H⋯N/N⋯H, H⋯C/C⋯H and H⋯O/O⋯H inter­actions in Fig. 8 ▸ *a*–*d*, respectively. The Hirshfeld surface analysis confirms the importance of H-atom contacts in establishing the packing. The large number of H⋯H, H⋯N/N⋯H, H ⋯ C/C⋯H and H⋯O/O⋯H inter­actions suggest that van der Waals inter­actions and hydrogen bonding play the major roles in the crystal packing (Hathwar *et al.*, 2015 ▸).

## 5. Crystal voids

**Figure 9:**
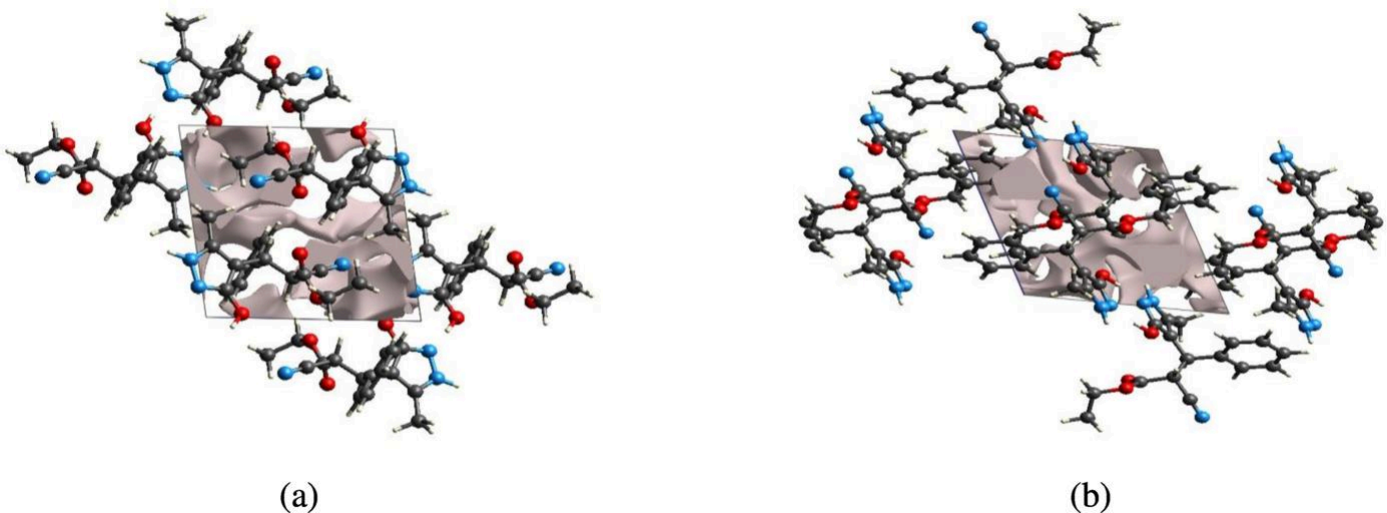
Graphical views of voids in the crystal packing of (I) (*a*) along the *a*-axis direction and (*b*) along the *b*-axis direction.

The strength of the crystal packing is important for determining the response to an applied mechanical force. If the crystal packing results in significant voids, then the mol­ecules are not tightly packed and a small amount of applied external mechanical force may easily break the crystal. A void analysis was performed to check the mechanical stability of the crystal by adding up the electron densities of the spherically symmetric atoms contained in the asymmetric unit (Turner *et al.*, 2011 ▸). The void surface is defined as an isosurface of the procrystal electron density and is calculated for the whole unit cell where the void surface meets the boundary of the unit cell and capping faces are generated to create an enclosed volume. The volume of the crystal voids (Fig. 9 ▸ *a*,*b*) and the percentage of free space in the unit cell are calculated as 100.94 Å^3^ and 13.20%, respectively. Thus, the crystal packing appears compact and the mechanical stability should be substantial.

## 6. Inter­action energy calculations and energy frameworks

The inter­molecular inter­action energies are calculated using the CE–B3LYP/6–31G(d,p) energy model available in *Crystal Explorer 17.5* (Turner *et al.*, 2017 ▸), where a cluster of mol­ecules is generated by applying crystallographic symmetry operations with respect to a selected central mol­ecule within the radius of 3.8 Å by default (Turner *et al.*, 2014 ▸). The total inter­molecular energy (*E*_tot_) is the sum of electrostatic (*E*_ele_),polarization (*E*_pol_), dispersion (*E*_dis_) and exchange-repulsion (*E*_rep_) energies (Turner *et al.*, 2015 ▸) with scale factors of 1.057, 0.740, 0.871 and 0.618, respectively (Mackenzie *et al.*, 2017 ▸). Hydrogen-bonding inter­action energies (in kJ mol^−1^) were calculated to be −141.9 (*E*_ele_), −31.4 (*E*_pol_), −19.8 (*E*_dis_), 174.8 (*E*_rep_) and −82.6 (*E*_tot_) for O3—H3⋯N2 and −23.3 (*E*_ele_), −3.5 (*E*_pol_), −50.6 (*E*_dis_), 26.4 (*E*_rep_) and −55.0 (*E*_tot_) for N1—H1⋯N3.

**Figure 10:**
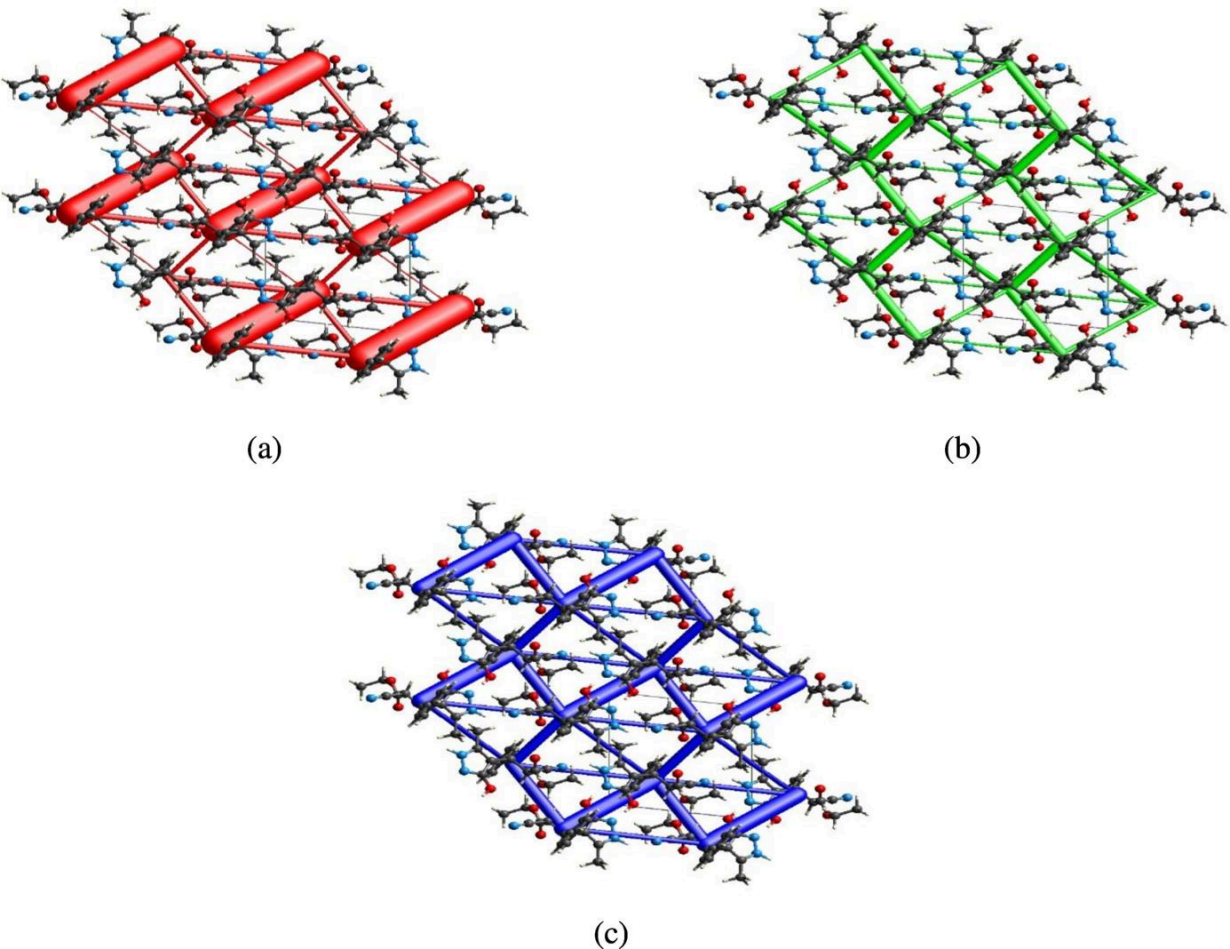
The energy frameworks for a cluster of mol­ecules of the title compound viewed down the *a*-axis direction showing (*a*) electrostatic energy, (*b*) dispersion energy and (*c*) total energy diagrams. The cylindrical radius is proportional to the relative strength of the corresponding energies and they were adjusted to the same scale factor of 80 with cut-off value of 5 kJ mol^−1^ within 2 × 2 × 2 unit cells.

Energy frameworks combine the calculation of inter­molecular inter­action energies with a graphical representation of their magnitude (Turner *et al.*, 2015 ▸). Energies between mol­ecular pairs are represented as cylinders joining the centroids of pairs of mol­ecules with the cylinder radius proportional to the relative strength of the corresponding inter­action energy. Energy frameworks were constructed for *E*_ele_ (red cylinders), *E*_dis_ (green cylinders) and *E*_tot_ (blue cylinders) (Fig. 10 ▸ *a*,*b*,*c*). The evaluation of the electrostatic, dispersion and total energy frameworks indicate that the stabilization is dominated by the electrostatic energy contribution in the crystal structure of (I).

## 7. Database survey

**Figure 11:**
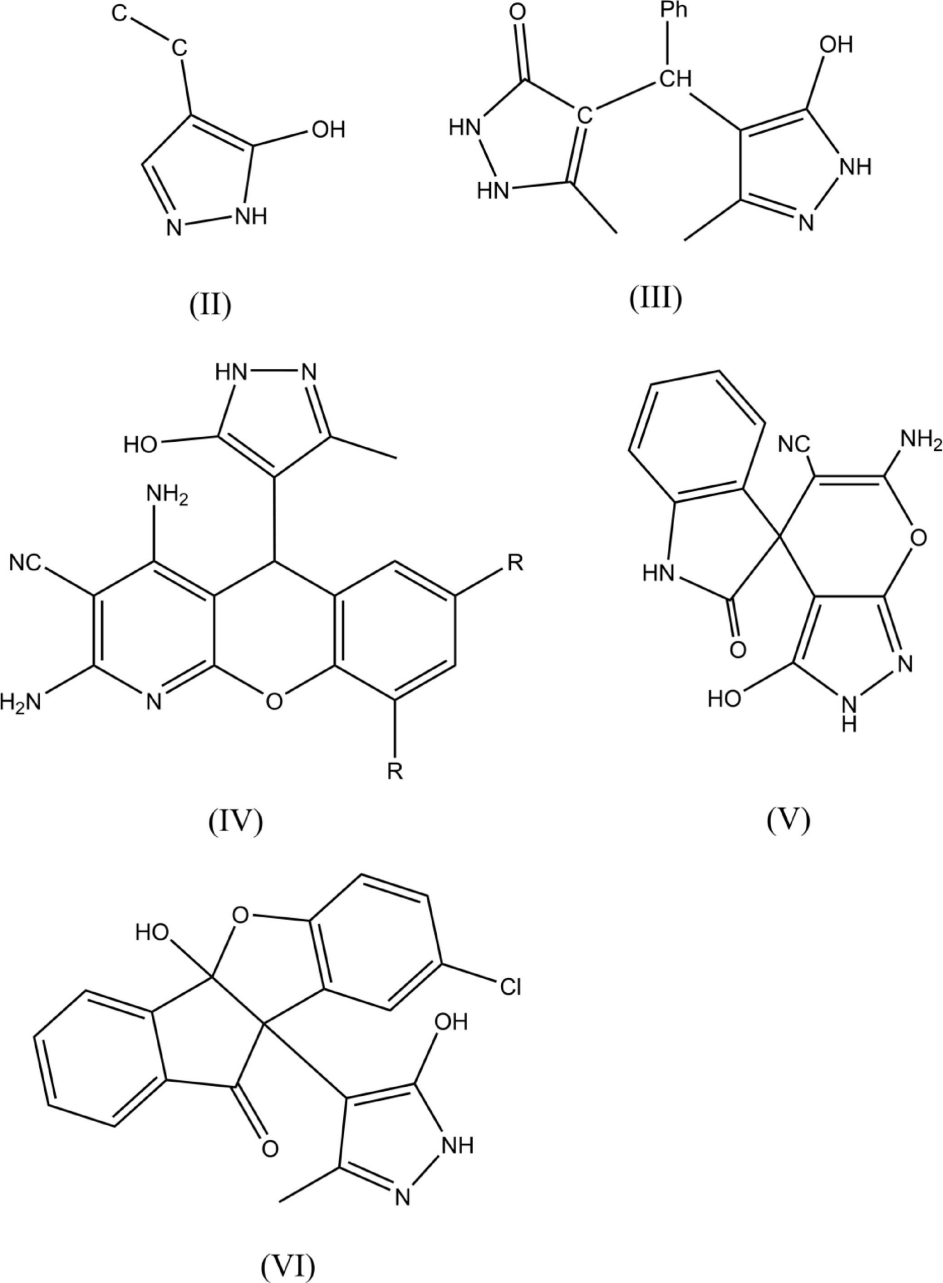
The closest matches to the title compound (I) according to the results obtained from the database survey.

A search of the Cambridge Structural Database (Groom *et al.*, 2016 ▸; updated to November 2023) located no other structures similar to (I) until the search fragment was simplified to (II) (Fig. 11 ▸). With this, five hits were obtained with (III) (RUWZUH; Zonouz *et al.*, 2020 ▸) being the closest match. The others are (IV) (*R* = Cl, IDOGUG; Elinson *et al.*, 2018*a* ▸, *R* = H, FINWAD; Elinson *et al.*, 2018*b* ▸), (V) (GEXSUA; Moghadam, 2018 ▸) and (VI) (TIWGUD; Pathak *et al.*, 2013 ▸) (Fig. 11 ▸).

## 8. DFT calculations

**Figure 12:**
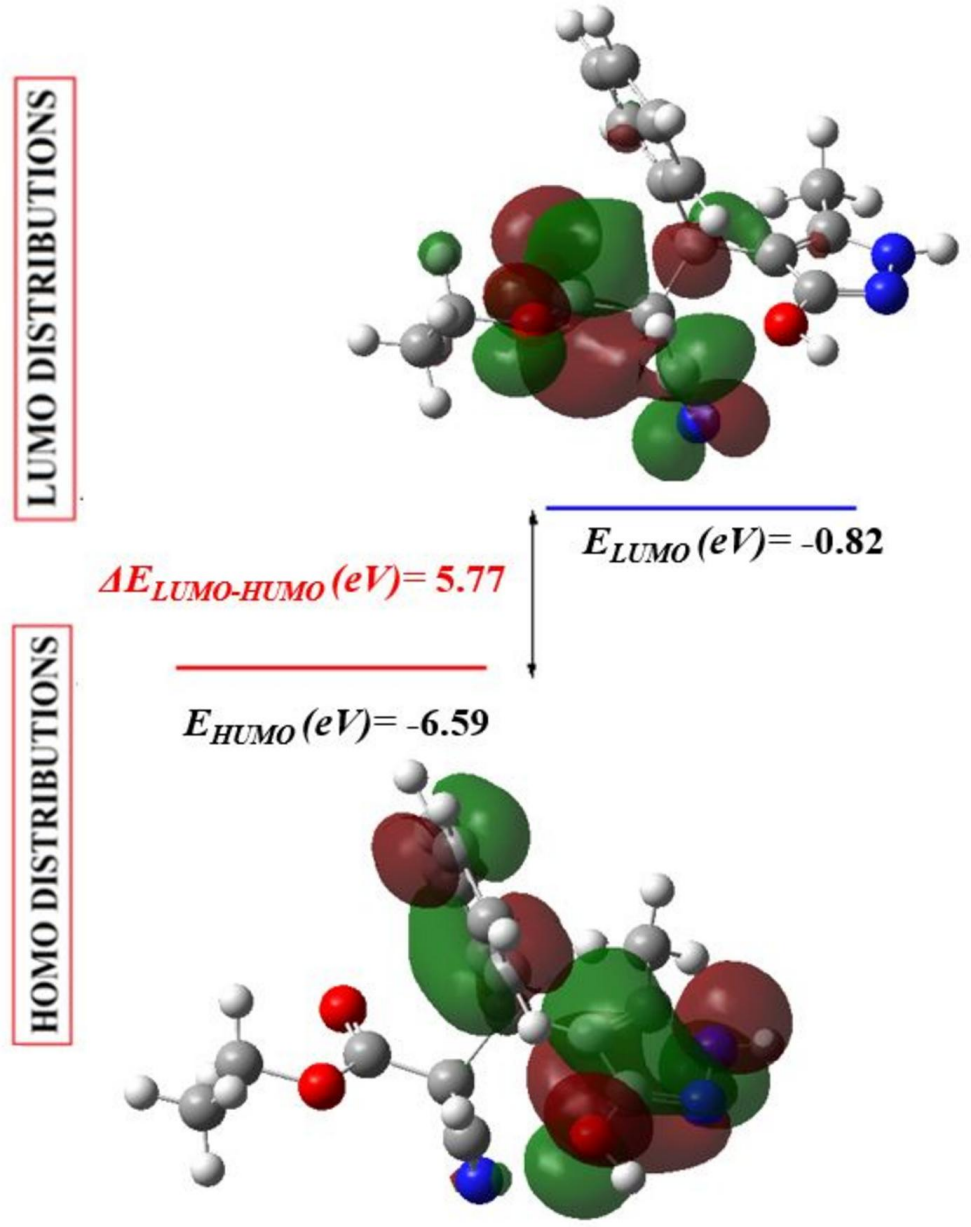
The energy band gap of the title compound.

The theoretical optimization of the mol­ecular structure in the gas-phase was carried out using density functional theory (DFT) with the standard B3LYP functional and 6-311G(d,p) basis-set calculations (Becke, 1993 ▸) as implemented in *GAUSSIAN 09* (Frisch *et al.*, 2009 ▸). The resulting optimized parameters (bond lengths and angles) agreed satisfactorily with the experimental structural data (Table 2 ▸). The largest differences between the calculated and experimental values are observed for the O1—C2 (0.06 Å) and O2—C3 (0.03 Å) bond lengths and the C3—O1—C2 and N2—C6—O3 bond angle (1.07°). These disparities can be linked to the fact that these calculations relate to the isolated mol­ecule, whereas the experimental results correspond to inter­acting mol­ecules in the crystal where intra- and inter­molecular inter­actions with neighbouring mol­ecules are present. The highest-occupied mol­ecular orbital (HOMO), acting as an electron donor, and the lowest-unoccupied mol­ecular orbital (LUMO), acting as an electron acceptor, are very important parameters for quantum chemistry. When the energy gap is small, the mol­ecule is highly polarizable and has high chemical reactivity (Elmachkouri *et al.*, 2023*a* ▸). The numerical reactivity descriptors (ionization potential, electron affinity, chemical hardness, chemical softness, electronegativity, chemical potential, electrophilicity index and total energy), which are mainly based on the HOMO–LUMO energies, are summarized in Table 3 ▸. The optimized frontier mol­ecular orbitals (HOMO and LUMO) are shown in Fig. 12 ▸. The LUMO is mainly centered on the 2-cyano group and spans the entire ethyl propano­ate chain while the HOMO is primarily centered on the 3-phenyl substituent and spans the 3-(3-hy­droxy-5-methyl-1*H*-pyrazol-4-yl) portion. The energy band gap [(*E* = *E*_LUMO_ - *E*_HOMO_) of the mol­ecule is about 5.77 eV, and the frontier mol­ecular orbital energies, *E*_HOMO_ and *E*_LUMO_, are −6.59 eV and −0.82eV, respectively.

## 9. Synthesis and crystallization

To a solution of pyrazolone (4 mmol), benzaldehyde (4 mmol) and ethyl 2-cyano­acetate (4 mmol, 0.42 ml) in absolute ethanol (12 ml), were added two drops of piperidine and the reaction mixture was refluxed with magnetic stirring for 2 h. The progress of the reaction was monitored by TLC using an ethyl acetate/hexane mixture as eluant. Finally, the resulting precipitate was filtered and the isolated solid was purified by recrystallization from ethanol to afford colourless crystals in 96% yield. The melting point was 454 K.

## 10. Refinement

Crystal data, data collection and structure refinement details are summarized in Table 4 ▸. Hydrogen atoms attached to carbon were included as riding contributions in idealized positions with isotropic displacement parameters tied to those of the attached atoms while those attached to nitro­gen and to oxygen were located in a difference map and refined with DFIX 0.91 0.01 and DFIX 0.85 0.01 instructions, respectively.

## Supplementary Material

Crystal structure: contains datablock(s) global, I. DOI: 10.1107/S2056989024000744/ny2002sup1.cif

Structure factors: contains datablock(s) I. DOI: 10.1107/S2056989024000744/ny2002Isup2.hkl

Supporting information file. DOI: 10.1107/S2056989024000744/ny2002Isup3.cdx

Supporting information file. DOI: 10.1107/S2056989024000744/ny2002Isup4.cml

CCDC reference: 2327436

Additional supporting information:  crystallographic information; 3D view; checkCIF report

## Figures and Tables

**Table 1:** Hydrogen-bond geometry (Å, °) *Cg*1 is the centroid of the five-membered ring.

*D*—H⋯*A*	*D*—H	H⋯*A*	*D*⋯*A*	*D*—H⋯*A*
O3—H3⋯N2^i^	0.86 (1)	1.85 (1)	2.706 (2)	175 (3)
N1—H1⋯N3^ii^	0.91 (1)	2.13 (2)	2.948 (2)	149 (2)
C14—H14⋯*Cg*1^iii^	0.95	2.71	3.627 (3)	162

Symmetry codes: (i) 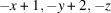 ; (ii) 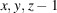 ; (iii) 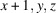 .

**Table 2:** Comparison of selected X-ray and DFT geometric data (Å, °)

Bonds/angles	X-ray	B3LYP/6–311G(d,p)
O1—C3	1.335 (2)	1.345
O1—C2	1.460 (3)	1.521
O2—C3	1.204 (2)	1.235
O3—C6	1.340 (2)	1.337
N1—C8	1.348 (3)	1.331
N1—N2	1.368 (2)	1.377
N1—H1	0.914 (10)	0.87
N2—C6	1.335 (2)	1.322
N3—C5	1.141 (3)	1.128
C3—O1—C2	116.67 (17)	115.6
C8—N1—N2	112.35 (16)	113.1
C6—N2—N1	103.86 (16)	104.6
O2—C3—O1	125.51 (19)	124.5
N2—C6—O3	122.03 (18)	123.1

**Table 3:** Calculated energies

Mol­ecular energy (a.u.) (eV)	Compound (I)
*E*_LUMO_ (eV)	−0.82
*E*_HOMO_ (eV)	−6.59
Gap *ΔE* (eV)	5.77
Ionization potential *I*	6.59
Electron affinity *A*	0.82
Chemical hardness *η*	2.88
Chemical softness *σ*	0.17
Electronegativity *χ*	3.70
Chemical potential *μ*	−3.71
Electrophilicity index *ω*	2.38
Total energy *TE* (eV)	−27476.33

**Table 4:** Experimental details

Crystal data	
Chemical formula	C_16_H_17_N_3_O_3_
*M*_r_	299.32
Crystal system, space group	Triclinic, *P* 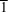
Temperature (K)	150
*a*, *b*, *c* (Å)	9.1397 (2), 9.4879 (2), 10.0063 (2)
α, β, γ (°)	79.554 (1), 63.787 (1), 83.054 (1)
*V* (Å^3^)	764.75 (3)
*Z*	2
Radiation type	Cu *K*α
μ (mm^−1^)	0.75
Crystal size (mm)	0.16 × 0.09 × 0.03

Data collection	
Diffractometer	Bruker D8 VENTURE PHOTON 3 CPAD
Absorption correction	Multi-scan (*SADABS*; Krause *et al.*, 2015 ▸)
*T*_min_, *T*_max_	0.88, 0.98
No. of measured, independent and observed [*I* > 2σ(*I*)] reflections	36885, 2998, 2207
*R*_int_	0.112
(sin θ/λ)_max_ (Å^−1^)	0.618

Refinement	
*R*[*F* ^2^ > 2σ(*F* ^2^)], *wR*(*F* ^2^), *S*	0.048, 0.127, 1.04
No. of reflections	2998
No. of parameters	207
No. of restraints	2
H-atom treatment	H atoms treated by a mixture of independent and constrained refinement
Δρ_max_, Δρ_min_ (e Å^−3^)	0.19, −0.23

Computer programs: *APEX4* and *SAINT* (Bruker, 2021 ▸), *SHELXT* (Sheldrick, 2015*a* ▸), *SHELXL2019/1* (Sheldrick, 2015*b* ▸), *DIAMOND* (Brandenburg & Putz, 2012 ▸) and *SHELXTL* (Sheldrick, 2008).

## supplementary crystallographic information

### Crystal data

**Table:**

C_16_H_17_N_3_O_3_	*Z* = 2
*M**_r_* = 299.32	*F*(000) = 316
Triclinic, *P*1	*D*_x_ = 1.300 Mg m^−^^3^
*a* = 9.1397 (2) Å	Cu *K*α radiation, λ = 1.54178 Å
*b* = 9.4879 (2) Å	Cell parameters from 8141 reflections
*c* = 10.0063 (2) Å	θ = 4.7–72.2°
α = 79.554 (1)°	µ = 0.75 mm^−^^1^
β = 63.787 (1)°	*T* = 150 K
γ = 83.054 (1)°	Prism, clear colourless
*V* = 764.75 (3) Å^3^	0.16 × 0.09 × 0.03 mm

### Data collection

**Table:**

Bruker D8 VENTURE PHOTON 3 CPAD diffractometer	2998 independent reflections
Radiation source: INCOATEC IµS micro–focus source	2207 reflections with *I* > 2σ(*I*)
Mirror monochromator	*R*_int_ = 0.112
Detector resolution: 7.3910 pixels mm^-1^	θ_max_ = 72.4°, θ_min_ = 4.7°
φ and ω scans	*h* = −11→11
Absorption correction: multi-scan (*SADABS*; Krause *et al.*, 2015)	*k* = −11→11
*T*_min_ = 0.88, *T*_max_ = 0.98	*l* = −12→12
36885 measured reflections	

### Refinement

**Table:**

Refinement on *F*^2^	Primary atom site location: dual
Least-squares matrix: full	Secondary atom site location: difference Fourier map
*R*[*F*^2^ > 2σ(*F*^2^)] = 0.048	Hydrogen site location: mixed
*wR*(*F*^2^) = 0.127	H atoms treated by a mixture of independent and constrained refinement
*S* = 1.04	*w* = 1/[σ^2^(*F*_o_^2^) + (0.0565*P*)^2^ + 0.3004*P*] where *P* = (*F*_o_^2^ + 2*F*_c_^2^)/3
2998 reflections	(Δ/σ)_max_ < 0.001
207 parameters	Δρ_max_ = 0.19 e Å^−^^3^
2 restraints	Δρ_min_ = −0.23 e Å^−^^3^

### Special details

**Table:**

Experimental. The diffraction data were obtained from sets of frames, each of width 0.5° in ω or φ, collected with scan parameters determined by the "strategy" routine in *APEX4*. The scan time was sec/frame.
Geometry. All esds (except the esd in the dihedral angle between two l.s. planes) are estimated using the full covariance matrix. The cell esds are taken into account individually in the estimation of esds in distances, angles and torsion angles; correlations between esds in cell parameters are only used when they are defined by crystal symmetry. An approximate (isotropic) treatment of cell esds is used for estimating esds involving l.s. planes.
Refinement. Refinement of F^2^ against ALL reflections. The weighted R-factor wR and goodness of fit S are based on F^2^, conventional R-factors R are based on F, with F set to zero for negative F^2^. The threshold expression of F^2^ > 2sigma(F^2^) is used only for calculating R-factors(gt) etc. and is not relevant to the choice of reflections for refinement. R-factors based on F^2^ are statistically about twice as large as those based on F, and R- factors based on ALL data will be even larger. H-atoms attached to carbon were placed in calculated positions (C—H = 0.95 - 1.00 Å) and were included as riding contributions with isotropic displacement parameters 1.2 - 1.5 times those of the attached atoms. Those attached to nitrogen and to oxygen were placed in locations derived from a difference map and refined with DFIX 0.91 0.01 and DFIX 0.85 0.01 instructions, respectively.

### Fractional atomic coordinates and isotropic or equivalent isotropic displacement parameters (Å^2^)

**Table:**

	*x*	*y*	*z*	*U*_iso_*/*U*_eq_	
O1	0.31072 (17)	0.88849 (16)	0.53196 (17)	0.0374 (4)	
O2	0.34195 (18)	0.66225 (16)	0.47923 (18)	0.0396 (4)	
O3	0.5608 (2)	0.97967 (16)	0.16765 (17)	0.0380 (4)	
H3	0.510 (3)	1.038 (2)	0.124 (3)	0.057*	
N1	0.6348 (2)	0.70329 (19)	−0.03454 (19)	0.0340 (4)	
H1	0.641 (3)	0.670 (3)	−0.1172 (19)	0.051*	
N2	0.5886 (2)	0.84391 (19)	−0.01623 (19)	0.0327 (4)	
N3	0.6355 (2)	0.7145 (2)	0.6688 (2)	0.0441 (5)	
C1	0.0507 (3)	0.8091 (3)	0.7386 (3)	0.0574 (7)	
H1A	−0.067805	0.824670	0.774657	0.086*	
H1B	0.085342	0.844719	0.805661	0.086*	
H1C	0.079772	0.706292	0.737677	0.086*	
C2	0.1346 (3)	0.8881 (3)	0.5822 (3)	0.0453 (6)	
H2A	0.112275	0.842376	0.512074	0.054*	
H2B	0.089410	0.988276	0.579737	0.054*	
C3	0.3974 (2)	0.7698 (2)	0.4823 (2)	0.0306 (4)	
C4	0.5793 (2)	0.7876 (2)	0.4307 (2)	0.0278 (4)	
H4	0.604261	0.890474	0.389034	0.033*	
C5	0.6124 (2)	0.7486 (2)	0.5641 (2)	0.0333 (5)	
C6	0.5994 (2)	0.8562 (2)	0.1099 (2)	0.0301 (4)	
C7	0.6532 (2)	0.7267 (2)	0.1720 (2)	0.0278 (4)	
C8	0.6750 (2)	0.6317 (2)	0.0746 (2)	0.0316 (4)	
C9	0.7316 (3)	0.4776 (2)	0.0780 (3)	0.0443 (6)	
H9A	0.680821	0.428399	0.032257	0.066*	
H9B	0.700504	0.432922	0.182580	0.066*	
H9C	0.850573	0.470104	0.021522	0.066*	
C10	0.6881 (2)	0.6924 (2)	0.3084 (2)	0.0277 (4)	
H10	0.660923	0.590388	0.352463	0.033*	
C11	0.8671 (2)	0.7065 (2)	0.2704 (2)	0.0276 (4)	
C12	0.9482 (2)	0.5996 (2)	0.3305 (2)	0.0335 (5)	
H12	0.892943	0.516295	0.391447	0.040*	
C13	1.1095 (3)	0.6133 (3)	0.3023 (2)	0.0391 (5)	
H13	1.163765	0.539442	0.343921	0.047*	
C14	1.1906 (3)	0.7340 (3)	0.2139 (3)	0.0410 (5)	
H14	1.300235	0.744139	0.195775	0.049*	
C15	1.1118 (3)	0.8403 (3)	0.1519 (3)	0.0399 (5)	
H15	1.168056	0.922894	0.090037	0.048*	
C16	0.9514 (3)	0.8269 (2)	0.1793 (2)	0.0356 (5)	
H16	0.898446	0.900196	0.135782	0.043*	

### Atomic displacement parameters (Å^2^)

**Table:**

	*U*^11^	*U*^22^	*U*^33^	*U*^12^	*U*^13^	*U*^23^
O1	0.0302 (8)	0.0378 (8)	0.0425 (9)	0.0015 (6)	−0.0144 (6)	−0.0070 (7)
O2	0.0342 (8)	0.0396 (9)	0.0476 (9)	−0.0058 (6)	−0.0179 (7)	−0.0090 (7)
O3	0.0555 (10)	0.0341 (8)	0.0356 (8)	0.0037 (7)	−0.0318 (7)	−0.0034 (6)
N1	0.0400 (10)	0.0407 (10)	0.0253 (8)	−0.0016 (7)	−0.0176 (7)	−0.0055 (7)
N2	0.0360 (9)	0.0390 (10)	0.0265 (8)	−0.0032 (7)	−0.0173 (7)	−0.0013 (7)
N3	0.0489 (12)	0.0569 (13)	0.0323 (10)	0.0084 (9)	−0.0241 (9)	−0.0097 (9)
C1	0.0411 (14)	0.0526 (16)	0.0616 (17)	−0.0039 (11)	−0.0058 (12)	−0.0099 (13)
C2	0.0330 (12)	0.0523 (14)	0.0522 (14)	0.0081 (10)	−0.0198 (10)	−0.0138 (11)
C3	0.0314 (10)	0.0362 (11)	0.0256 (10)	−0.0001 (8)	−0.0145 (8)	−0.0027 (8)
C4	0.0304 (10)	0.0312 (10)	0.0241 (9)	−0.0026 (8)	−0.0148 (8)	−0.0003 (8)
C5	0.0320 (11)	0.0404 (12)	0.0294 (10)	0.0002 (8)	−0.0150 (9)	−0.0061 (9)
C6	0.0312 (10)	0.0385 (11)	0.0245 (9)	−0.0039 (8)	−0.0154 (8)	−0.0035 (8)
C7	0.0264 (9)	0.0358 (11)	0.0218 (9)	−0.0037 (7)	−0.0117 (7)	−0.0004 (8)
C8	0.0333 (10)	0.0367 (11)	0.0257 (9)	−0.0043 (8)	−0.0143 (8)	−0.0011 (8)
C9	0.0606 (15)	0.0409 (13)	0.0370 (12)	0.0051 (11)	−0.0265 (11)	−0.0095 (10)
C10	0.0319 (10)	0.0275 (10)	0.0264 (9)	−0.0037 (7)	−0.0161 (8)	0.0004 (8)
C11	0.0291 (10)	0.0324 (10)	0.0232 (9)	−0.0018 (8)	−0.0127 (8)	−0.0041 (8)
C12	0.0349 (11)	0.0376 (12)	0.0291 (10)	−0.0014 (9)	−0.0160 (9)	−0.0017 (9)
C13	0.0340 (11)	0.0511 (14)	0.0359 (11)	0.0050 (9)	−0.0204 (9)	−0.0054 (10)
C14	0.0293 (11)	0.0595 (15)	0.0378 (12)	−0.0053 (10)	−0.0152 (9)	−0.0119 (10)
C15	0.0380 (12)	0.0437 (13)	0.0374 (12)	−0.0119 (10)	−0.0146 (9)	−0.0021 (10)
C16	0.0359 (11)	0.0360 (12)	0.0346 (11)	−0.0048 (9)	−0.0167 (9)	0.0019 (9)

### Geometric parameters (Å, º)

**Table:**

O1—C3	1.335 (2)	C6—C7	1.409 (3)
O1—C2	1.460 (3)	C7—C8	1.380 (3)
O2—C3	1.204 (2)	C7—C10	1.506 (3)
O3—C6	1.340 (2)	C8—C9	1.490 (3)
O3—H3	0.857 (10)	C9—H9A	0.9800
N1—C8	1.348 (3)	C9—H9B	0.9800
N1—N2	1.370 (2)	C9—H9C	0.9800
N1—H1	0.914 (10)	C10—C11	1.524 (3)
N2—C6	1.335 (2)	C10—H10	1.0000
N3—C5	1.141 (3)	C11—C12	1.389 (3)
C1—C2	1.501 (4)	C11—C16	1.394 (3)
C1—H1A	0.9800	C12—C13	1.391 (3)
C1—H1B	0.9800	C12—H12	0.9500
C1—H1C	0.9800	C13—C14	1.379 (3)
C2—H2A	0.9900	C13—H13	0.9500
C2—H2B	0.9900	C14—C15	1.383 (3)
C3—C4	1.529 (3)	C14—H14	0.9500
C4—C5	1.470 (3)	C15—C16	1.385 (3)
C4—C10	1.552 (3)	C15—H15	0.9500
C4—H4	1.0000	C16—H16	0.9500
			
C3—O1—C2	116.68 (17)	N1—C8—C7	107.38 (18)
C6—O3—H3	109.6 (19)	N1—C8—C9	122.35 (18)
C8—N1—N2	112.28 (16)	C7—C8—C9	130.27 (18)
C8—N1—H1	127.6 (17)	C8—C9—H9A	109.5
N2—N1—H1	120.0 (17)	C8—C9—H9B	109.5
C6—N2—N1	103.85 (16)	H9A—C9—H9B	109.5
C2—C1—H1A	109.5	C8—C9—H9C	109.5
C2—C1—H1B	109.5	H9A—C9—H9C	109.5
H1A—C1—H1B	109.5	H9B—C9—H9C	109.5
C2—C1—H1C	109.5	C7—C10—C11	112.75 (16)
H1A—C1—H1C	109.5	C7—C10—C4	111.51 (16)
H1B—C1—H1C	109.5	C11—C10—C4	109.52 (15)
O1—C2—C1	111.9 (2)	C7—C10—H10	107.6
O1—C2—H2A	109.2	C11—C10—H10	107.6
C1—C2—H2A	109.2	C4—C10—H10	107.6
O1—C2—H2B	109.2	C12—C11—C16	118.64 (18)
C1—C2—H2B	109.2	C12—C11—C10	119.87 (17)
H2A—C2—H2B	107.9	C16—C11—C10	121.46 (18)
O2—C3—O1	125.49 (19)	C11—C12—C13	120.77 (19)
O2—C3—C4	123.93 (19)	C11—C12—H12	119.6
O1—C3—C4	110.57 (17)	C13—C12—H12	119.6
C5—C4—C3	107.30 (16)	C14—C13—C12	120.0 (2)
C5—C4—C10	110.20 (16)	C14—C13—H13	120.0
C3—C4—C10	112.11 (15)	C12—C13—H13	120.0
C5—C4—H4	109.1	C13—C14—C15	119.8 (2)
C3—C4—H4	109.1	C13—C14—H14	120.1
C10—C4—H4	109.1	C15—C14—H14	120.1
N3—C5—C4	177.9 (2)	C14—C15—C16	120.4 (2)
N2—C6—O3	122.06 (18)	C14—C15—H15	119.8
N2—C6—C7	112.33 (18)	C16—C15—H15	119.8
O3—C6—C7	125.61 (17)	C15—C16—C11	120.4 (2)
C8—C7—C6	104.16 (17)	C15—C16—H16	119.8
C8—C7—C10	125.22 (18)	C11—C16—H16	119.8
C6—C7—C10	130.58 (18)		
			
C8—N1—N2—C6	−0.9 (2)	C8—C7—C10—C11	83.8 (2)
C3—O1—C2—C1	79.9 (2)	C6—C7—C10—C11	−93.6 (2)
C2—O1—C3—O2	−1.9 (3)	C8—C7—C10—C4	−152.51 (19)
C2—O1—C3—C4	178.73 (17)	C6—C7—C10—C4	30.1 (3)
O2—C3—C4—C5	−93.5 (2)	C5—C4—C10—C7	178.19 (16)
O1—C3—C4—C5	85.9 (2)	C3—C4—C10—C7	58.8 (2)
O2—C3—C4—C10	27.6 (3)	C5—C4—C10—C11	−56.3 (2)
O1—C3—C4—C10	−152.99 (16)	C3—C4—C10—C11	−175.76 (15)
N1—N2—C6—O3	−178.86 (18)	C7—C10—C11—C12	−133.87 (19)
N1—N2—C6—C7	0.7 (2)	C4—C10—C11—C12	101.4 (2)
N2—C6—C7—C8	−0.2 (2)	C7—C10—C11—C16	47.9 (3)
O3—C6—C7—C8	179.32 (19)	C4—C10—C11—C16	−76.9 (2)
N2—C6—C7—C10	177.66 (19)	C16—C11—C12—C13	1.0 (3)
O3—C6—C7—C10	−2.8 (3)	C10—C11—C12—C13	−177.28 (18)
N2—N1—C8—C7	0.9 (2)	C11—C12—C13—C14	0.0 (3)
N2—N1—C8—C9	−179.08 (19)	C12—C13—C14—C15	−0.9 (3)
C6—C7—C8—N1	−0.4 (2)	C13—C14—C15—C16	0.7 (3)
C10—C7—C8—N1	−178.39 (17)	C14—C15—C16—C11	0.3 (3)
C6—C7—C8—C9	179.5 (2)	C12—C11—C16—C15	−1.2 (3)
C10—C7—C8—C9	1.5 (3)	C10—C11—C16—C15	177.08 (19)

### Hydrogen-bond geometry (Å, º)

*Cg*1 is the centroid of the five-membered ring.

**Table:**

*D*—H···*A*	*D*—H	H···*A*	*D*···*A*	*D*—H···*A*
O3—H3···N2^i^	0.86 (1)	1.85 (1)	2.706 (2)	175 (3)
N1—H1···N3^ii^	0.91 (1)	2.13 (2)	2.948 (2)	149 (2)
C14—H14···*Cg*1^iii^	0.95	2.71	3.627 (3)	162

Symmetry codes: (i) −*x*+1, −*y*+2, −*z*; (ii) *x*, *y*, *z*−1; (iii) *x*+1, *y*, *z*.
